# Dose-Response Associations of Metabolic Score for Insulin Resistance Index with Nonalcoholic Fatty Liver Disease among a Nonobese Chinese Population: Retrospective Evidence from a Population-Based Cohort Study

**DOI:** 10.1155/2022/4930355

**Published:** 2022-02-23

**Authors:** Xintian Cai, Jing Gao, Junli Hu, Wen Wen, Qing Zhu, Mengru Wang, Shasha Liu, Jing Hong, Ting Wu, Shunfan Yang, Guzailinuer Tuerxun, Nanfang Li

**Affiliations:** ^1^Hypertension Center of People's Hospital of Xinjiang Uygur Autonomous Region, Xinjiang Hypertension Institute, National Health Committee Key Laboratory of Hypertension Clinical Research, Key Laboratory of Xinjiang Uygur Autonomous Region, Xinjiang Clinical Medical Research Center for Hypertension Diseases, Urumqi, China; ^2^Xinjiang Medical University, Urumqi, China; ^3^Research and Education Center of Xinjiang Uygur Autonomous Region People's Hospital, Urumqi, China

## Abstract

**Purpose:**

This study is aimed at investigating the association between the metabolic score for insulin resistance (METS-IR) index and nonalcoholic fatty liver disease (NAFLD) in the nonobese population and its predictive value.

**Methods:**

10730 nonobese subjects were selected from longitudinal cohort research conducted from January 2010 to December 2014. Cox proportional hazards models were employed to assess the relationship between METS-IR and new-onset NAFLD. Generalized additive models were used to identify nonlinear relationships. In addition, we performed subgroup analyses and interaction tests. The time-dependent receiver operating curve (ROC) and area under the ROC (AUC) were utilized to measure the discriminatory ability of METS-IR for new-onset NAFLD. Beyond clinical risk factors, the incremental predictive value of METS-IR was appraised using integrated discrimination improvement (IDI), C-index, and net reclassification index (NRI).

**Results:**

Over a median period of 804.50 days of follow-up, 1859 (17.33%) participants had a new onset of NAFLD. After adjusting for confounders, the HR for new-onset NAFLD in the Q4 group was 6.40 compared with the Q1 group. When METS-IR was considered a continuous variable, the risk of NAFLD increased by 34% for every 1 SD increase in METS-IR. The smoothing curve shows the dose-response relationship between METS-IR and the presence of new-onset NAFLD. Using a two-piecewise linear regression model, we derived a METS-IR inflection point of 36. HRs were 1.31 on the left side of the inflection point and 1.04 on the right side of the inflection point (log-likelihood ratio test, *P* < 0.001). Subgroup analyses and interaction tests revealed an interaction between gender and SBP in the association between METS-IR and new-onset NAFLD. In the subgroup analysis of gender and SBP, we observed a higher risk of new-onset NAFLD in men and in those with abnormal SBP levels. We evaluated the ability of METS-IR to identify new-onset NAFLD at different time points. The AUCs at 1, 2, 3, and 4 years were 0.784, 0.756, 0.758, and 0.752, respectively, which represent good discrimination of new-onset NAFLD. The addition of METS-IR greatly improved the reclassification and differentiation of clinical risk factors, with an NRI of 0.276 and an IDI of 0.068. In addition, the addition of METS-IR increased the C-index from 0.719 to 0.771.

**Conclusion:**

In a nonobese Chinese population, elevated METS-IR was independently associated with an enhanced risk of NAFLD development and a dose-response relationship existed. In addition, METS-IR might be a reliable indicator for screening individuals at risk for early NAFLD, especially in nonobese populations.

## 1. Introduction

Nonalcoholic fatty liver disease (NAFLD) is the predominant liver disease worldwide and a primary contributor to the development of diverse chronic hepatic disorders [1–3]. The prevalence of NAFLD in adults is reported to be about 25% globally [4]. In Asia, approximately 30% of individuals in the adult population are impacted by NAFLD, and this is growing by the day with the obesity epidemic [5, 6]. The potential burden of disease in NAFLD is going to exert substantial pressure on the safety of the medical and socioeconomic systems. Since no effective medications have been approved for the treatment of NAFLD for the time being, prevention and treatment of NAFLD are still mainly by reducing weight and improving lifestyle habits [7].

It is well known that obesity is strongly associated with the incidence and severity of NAFLD [8–10]. Nevertheless, numerous researches in recent years have noted that nonobese people are also susceptible to NAFLD [11–13]. The global incidence of NAFLD in the population of nonobese people has reached 40%, indicating that nonobese NAFLD accounts for a significant portion of the chronic hepatic disease burden [14]. Furthermore, a few large cohort reports have recently revealed that nonobese NAFLD individuals have higher all-cause mortality and accelerated disease progression compared to obese NAFLD patients, despite their less severe phenotype of metabolism [15–17].

It is well known that insulin resistance (IR) is implicated in critical aspects of the pathogenesis of NAFLD [18–20]. The homeostasis model assessment for insulin resistance (HOMA-IR) index is the method that is most commonly employed to assess the degree of IR [21, 22]. However, insulin testing is expensive and poorly reproducible, making it difficult to use in large epidemiological studies. Therefore, a simpler and practical index is necessary to assess IR. In recent years, the metabolic score for insulin resistance (METS-IR) index has received increasing attention as a simple index of IR [23–25]. Considering that IR serves as an essential component in the pathogenesis of NAFLD in nonobese individuals, the researchers, therefore, hypothesized that METS-IR may be a good marker for predicting the development of NAFLD in nonobese populations. Thus, this study is aimed at investigating the association between METS-IR and NAFLD in the nonobese populations and its predictive value and providing novel insights for the management of NAFLD.

## 2. Methods

### 2.1. Data Source

Raw data for the present study were derived from the Dryad database. Owing to the ownership of the raw data granted by Sun et al. [26] to the Dryad database, researchers were able to use the data for secondary analysis without violating the authors' rights. The source of the data was annotated in this study in accordance with the terms of usage of the database.

### 2.2. Study Design and Population

This study is a secondary analysis of data from a longitudinal cohort study. A previously published paper by Sun et al. demonstrates that the original study protocol was approved by the ethics committee of Wenzhou People's Hospital and that verbal informed consent was obtained from all participants. Participants in the original study were those who attended annual health checkups at Wenzhou People's Hospital between January 2010 and December 2014. In this longitudinal cohort study, 16173 nonobese individuals without NAFLD were initially enrolled. Exclusion criteria at baseline included (1) excessive alcohol consumption (>70 g/week for women or 140 g/week for men) (*n* = 3315), (2) taking lipid-lowering, antidiabetic, or blood pressure-lowering medications (*n* = 2272), (3) chronic liver disease due to other factors (*n* = 1492), (4) LDL-C > 3.12 mmol/L (*n* = 3320), (5) body mass index (BMI) ≥ 25 kg/m^2^ (*n* = 4260), (6) lost to follow-up (*n* = 2321), and (7) incomplete data (*n* = 5443). Ultimately, 10730 participants were incorporated into this analysis.

### 2.3. Data Acquisition and Follow-Up

All participants were instructed to avoid strenuous exercise the day before and to undergo a medical inspection in the all-night fasting morning. Trained medical personnel used standardized procedures to obtain participants' medical history and health habits. Blood pressure was monitored in a seated position using a standard electronic sphygmomanometer in a quiet environment, and systolic and diastolic blood pressures (S/DBP) were documented. Biochemical variables were measurable by automated analytical instruments. Details more specifically have been previously covered in reports. The biochemical parameters included in this study were as follows: total cholesterol (TC), fasting blood glucose (FPG), triglycerides (TG), alkaline phosphatase (ALP), low-density lipoprotein cholesterol (LDL-C), aspartate aminotransferase (AST), high-density lipoprotein cholesterol (HDL-C), total protein (TP), urea nitrogen (BUN), albumin (ALB), globulin (GLB), uric acid (UA), and creatinine (Cr).

The follow-up was initiated with the first NAFLD assessment of the subject by the clinician, followed by an annual NAFLD assessment by abdominal ultrasound. The maximum follow-up duration was 5 years.

### 2.4. Definitions

The diagnosis of NAFLD is made according to previously published diagnostic guidelines [27]. Briefly, the diagnostic criteria are to meet two of the following five abnormal echoes on abdominal ultrasonography, the first of which is essential for diagnosis: (1) diffuse hyperechogenicity relative to the spleen and kidney, (2) reduced visibility of detailed structures within the liver, (3) mild to moderate enlargement of the liver with bluntly rounded margins, (4) diminished hepatic blood flow signal with normal blood flow distribution, and (5) poorly defined or incomplete display of the right hepatic lobe and diaphragmatic envelope [27, 28]. METS-IR was calculated as (ln ((2 × FPG) + TG) × BMI)/(ln (HDL‐C)) [29].

### 2.5. Statistical Analysis

METS-IR was separated into four groups: 18.88-27.66, 27.66-30.93, 30.94-34.33, and 34.33-67.05. *χ*^2^ and Kruskal-Wallis tests were carried out to compare differences in categorical and continuous data among the four METS-IR categories.

The Kaplan-Meier method was applied to evaluate the cumulative incidence, and the log-rank test was utilized to examine the significance of the differences between groups. We incorporated all original covariates in an ordinary least squares model and tested for multicollinearity by calculating the variance inflation factor (VIF) for each covariate [30]. Covariates with VIF greater than 5 were deemed to have serious multicollinearity and could not be incorporated into the multiple regression model (Supplementary Table 1). Multivariate Cox proportional hazards models were employed to evaluate the association between METS-IR and new-onset NAFLD by generating hazard ratios (HRs) and 95% confidence intervals (CIs). To manipulate possible confounding bias, 2 models were developed for controlling baseline confounders: Model 1: age, BMI, and sex and Model 2: Model 1 plus ALP, TG, GGT, HDL-C, ALT, BUN, AST, Cr, UA, FPG, SBP, LDL-C, and DBP. A generalized additive model with a spline of smoothing was employed to examine the relationship between METS-IR and new-onset NAFLD.

A stratification analysis was further implemented to investigate the effects of potential modifications of the following factors: sex (women or men), age (<29, 30-59, or ≥60 years), BMI (<18.5 or ≥18.5 kg/m^2^), SBP (<140 or ≥140 mmHg), FPG (<6.1 or ≥6.1 mmol/L), DBP (<90 or ≥90 mmHg), UA (<416 or ≥416 mmol/L), and Cr (<104 or ≥104 *μ*mol/L). The impact of this modification was potentially evaluated by modeling the intersection product of stratification covariates with METS-IR.

The time-dependent receiver operating curve (ROC) was utilized to measure the ability of METS-IR to discriminate new-onset NAFLD. Beyond clinical risk factors, the incremental predictive value of METS-IR was appraised using the integrated discrimination improvement (IDI), C-index, and net reclassification index (NRI).

Statistical analysis concerned the utilization of R software (version 4.0.1). *P* < 0.05 (two-sided) was regarded as statistically significant.

## 3. Results

### 3.1. General Characteristics

**Figure 1:**
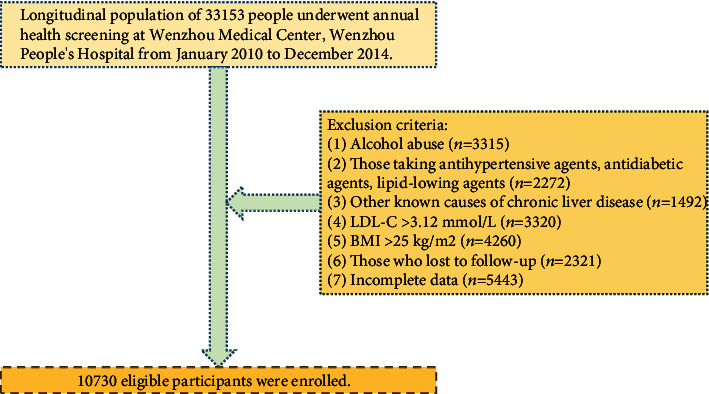
The study flowchart.

**Figure 2:**
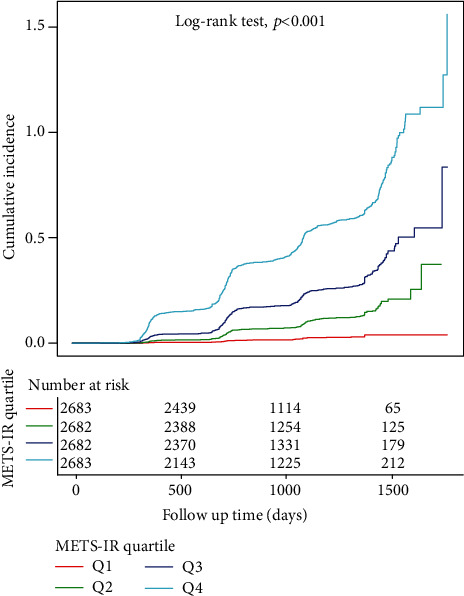
Kaplan-Meier curves of cumulative incidence of new-onset NAFLD stratified by METS-IR categories.

Of those initially invited, there were 10730 subjects who met the criteria for inclusion and finished follow-up who were enrolled in the analysis (Figure 1). Baseline clinical and biochemical features of the research population are outlined in Table 1. In general, the average age of the 10730 attendees was 43.65 years, and about 45.10% of the participants were female. Participants in the highest METS-IR group (Q4) had higher levels of BMI, SBP, age, FPG, DBP, GLB, AST, ALP, GGT, TP, BUN, TG, Cr, and UA in comparison to the remaining groups (Q1-3). During a median follow-up of 804.50 days (IQR, 703.00-1260.00), 1859 (17.33%) participants had new-onset NAFLD. The prevalence of NAFLD increased progressively with METS-IR (Q1: 1.49% vs. Q2: 7.72% vs. Q3: 19.31% vs. Q4: 40.78%). Furthermore, Kaplan-Meier curves demonstrated that participants in quartile 4 of METS-IR at baseline were at greater risk of incident during follow-up than participants in other groups (log-rank test, *P* < 0.001; Figure 2).

### 3.2. Association between METS-IR and New-Onset NAFLD

Table 2 and Supplementary Table 2 summarize outcomes of Cox regression analysis concerning the relationship between METS-IR and the new-onset NAFLD. After adjusting for sex, age, BMI, ALP, TG, GGT, HDL-C, ALT, BUN, AST, Cr, UA, FPG, SBP, LDL-C, and DBP, the HR for the new-onset NAFLD was 6.40 (95% CI: 4.06-10.08, *P* < 0.001) in Q4 versus Q1. In addition, there was a tendency for the new-onset NAFLD to increase with each of the METS-IR quartiles (*P* for trend < 0.001). When METS-IR was regarded as a continuous variable, the risk of NAFLD increased by 34% (95% CI: 1.04-1.73) for every 1 SD increase in METS-IR.

### 3.3. Dose-Response and Threshold Effect Analysis of METS-IR on New-Onset NAFLD

**Figure 3:**
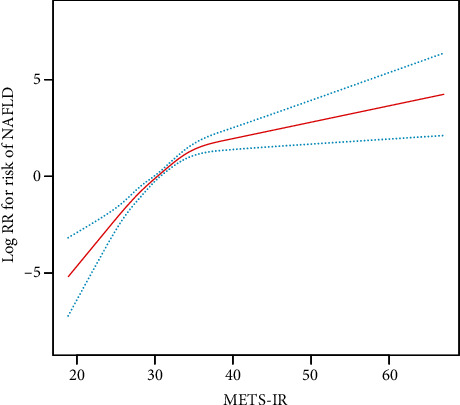
Dose-response relationship of METS-IR and new-onset NAFLD. The red line indicates the best-fit line, and the blue line represents the 95% confidence interval.

In Figure 3, the smoothed curve indicated the dose-response relationship between METS-IR and the presence of new-onset NAFLD. By using a two-piecewise linear regression model, we derived the inflection point of METS-IR to be 36. As illustrated in Table 3, the HRs were 1.31 (95% CI: 1.23-1.41) on the left side of the inflection point and 1.04 (95% CI: 0.98-1.10) on the right side of the inflection point (log-likelihood ratio test, *P* < 0.001).

### 3.4. Subgroup Analysis of METS-IR and New-Onset NAFLD

**Figure 4:**
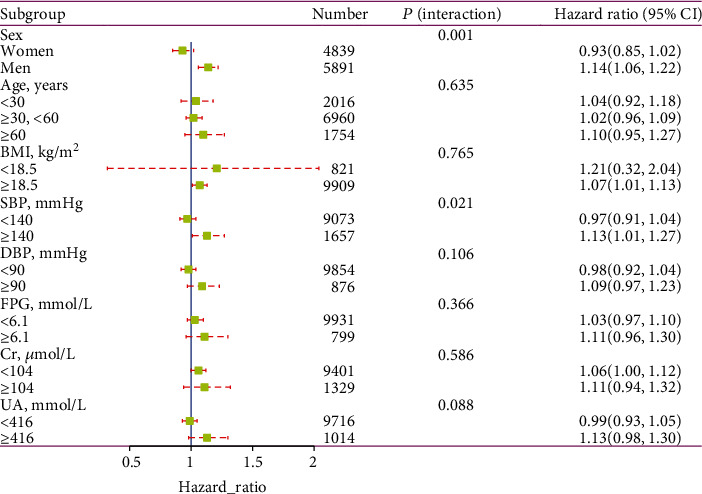
Association between METS-IR and new-onset NAFLD in various subgroups.

In the subgroup analysis, we investigated further the effect of other covariates on the relationship between METS-IR and new-onset NAFLD (Figure 4). Subgroup analysis and interaction tests detected that gender and SBP interacted in the association of METS-IR and new-onset NAFLD (all *P* for interactions < 0.05). In the subgroup analysis of gender and SBP, we observed a stronger risk of new-onset NAFLD in men (HR = 1.14, 95% CI: 1.06-1.22) and in those with abnormal SBP levels (SBP > 140 mmHg; HR = 1.13, 95% CI: 1.01-1.27).

### 3.5. Discriminative Power of METS-IR for New-Onset NAFLD

**Figure 5:**
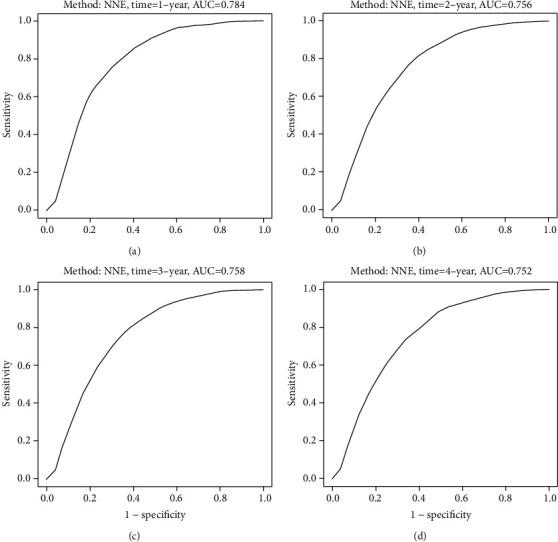
METS-IR is measured by the time-dependent receiver operating characteristic (ROC) curves at 1, 2, 3, and 4 years.

We appraised the discriminatory power of METS-IR for new-onset NAFLD at different time points (Figures 5(a)–5(d)). The AUC was 0.784 (95% CI: 0.773-0.794) at 1 year, 0.756 (95% CI: 0.746-0.767) at 2 years, 0.758 (95% CI: 0.747-0.768) at 3 years, and 0.752 (95% CI: 0.742-0.763), which represents a good discriminatory power for new-onset NAFLD.

### 3.6. Incremental Effect of METS-IR on Predictive Value for New-Onset NAFLD

Table 4 indicates that the addition of METS-IR dramatically improved the reclassification and differentiation of clinical risk factors with an NRI of 0.276 (0.247-0.303) and an IDI of 0.068 (0.057-0.080) (both *P* < 0.001). Additionally, the addition of METS-IR substantially increased the C-index from 0.719 (0.707-0.731) to 0.771 (0.761-0.781) (*P* < 0.001).

## 4. Discussion

With the economic boom and rapid acceptance of Western lifestyles in the Asia-Pacific region, NAFLD is a widespread condition among adults [31]. The prevalence of NAFLD in adults is approximately 20-30%, with nearly 10-25% of NAFLD patients progressing to nonalcoholic steatohepatitis and approximately 21-26% of nonalcoholic steatohepatitis patients progressing to cirrhosis within a few years [32, 33]. NAFLD not only induces a host of pathological changes in the liver but also contributes to the occurrence and development of a variety of extrahepatic diseases and has become an essential risk factor for a diverse range of metabolism-related diseases [34–38]. In spite of the fact that obesity is an influential risk factor for developing NAFLD, there is still a high prevalence of NAFLD in nonobese Asian populations [5, 14]. Therefore, there is a need to identify high-risk individuals in the nonobese Chinese population and to adopt specific preventive measures in advance.

To our knowledge, this is the first research to investigate the relationship between METS-IR index and new-onset NAFLD and to demonstrate the predictive effect of METS-IR on NAFLD in a nonobese Chinese population. This retrospective population-based cohort study revealed that participants with increased METS-IR in nonobese Chinese adults may have a higher cumulative incidence of NAFLD. And elevated METS-IR was independently associated with new-onset NAFLD. Compared with the lowest quartile, those in the highest METS-IR quartile had a 6.40-fold higher risk of new-onset NAFLD. Furthermore, this study not only assessed the independent effects of METS-IR and NAFLD risk but also addressed the dose-response association between them and calculated the inflection point of METS-IR at 36. When METS-IR < 36, METS-IR was significantly positively associated with the risk of NAFLD (HR = 1.31, 95% CI: 1.23-1.41, *P* < 0.001); however, when METS-IR ≥ 36, the trend gradually plateaued compared to the left side of the inflection point (HR = 1.04, 95% CI: 0.98-1.10, *P* = 0.169). In this study, factors including BMI, gender, age, DBP, SBP, FPG, Cr, and UA were used as stratifying variables, analyzed in subgroups, and tested for their interaction. A stronger association was observed in participants with SBP ≥ 140 mmHg and in male participants. Overall, based on the results of the subgroup analysis, METS-IR appears to be more sensitive in predicting NAFLD risk in men and individuals with abnormal SBP, suggesting that it may have promise for screening future NAFLD risk, especially in individuals with high-risk factors such as hypertension and men. More significantly, adding METS-IR to a baseline risk model consisting of certain clinical risk factors dramatically improves the ability to reclassify.

The mechanisms underlying the association of METS-IR with NAFLD in nonobese individuals have yet to be elucidated. First, IR performs a critical role in the development of NAFLD [39, 40]. METS-IR can be considered an independent predictor of IR [28]. As TG levels increase and HDL-C levels decrease, free fatty acids will increase with lipolysis [41]. An increase in free fatty acid levels can be brought about by a worsening of insulin sensitivity; inducing tissue oxidative stress will contribute to the development of insulin resistance in tissues [42–44]. Second, a variant allele of PNPLA3 (rs738409) is significantly increased in nonobese patients with NAFLD compared to healthy individuals. This variant allele is considered an independent risk factor in the nonobese NAFLD population [45, 46]. Wei et al. indicated that 78.4% of nonobese NAFLD patients harbored PNPLA3 (rs738409) [47]. The Dallas Heart Study identified PNPLA3 (rs738409) as being strongly associated with elevated hepatic TG levels and liver inflammation [48]. Besides, higher blood glucose levels can induce hepatotoxicity through activation of oxidative stress and endoplasmic reticulum stress response, directly leading to steatosis and hepatocyte death [49]. Chronic hyperglycemia can also provoke metabolic disturbances in the liver, promote mild inflammation, lead to IR, and trigger new lipid synthesis in the liver. IR, in turn, can further aggravate hepatic steatosis through lipotoxicity and inflammatory response [50]. Second, systemic low-grade inflammation exerts a central role in the pathogenesis of NAFLD. HDL-C exhibits anti-inflammatory functions by inhibiting the production of several proinflammatory cytokines and chemokines and by reducing the expression of adhesion molecules [51]. Third, oxidative stress is a well-known risk factor for the progression of NAFLD. HDL-C admits oxidized lipids and inhibits the oxidation of LDL-C [52]. Finally, stem cell growth factor-beta also has a major role in the mechanism of IR associated with male NAFLD patients [53].

The strength of this study is the population-based longitudinal cohort, study design, wide sample size, and reasonably tight adjustment for statistical covariates, which could interpret the causal relationship between METS-IR and new-onset NAFLD. Additionally, this research was the first to confirm a dose-response association between METS-IR and NAFLD. Regardless of all the advantages, there are a few possible vulnerabilities of the study. First, METS-IR and other biochemical parameters were measured only at initial enrollment, and the dynamic variations of these parameters during follow-up were not considered. Second, despite the fact that ultrasound screening is the widest recommended noninvasive diagnostic approach because of its great sensitivity and specificity, it is nonetheless less accurate compared with liver biopsy. Notably, abdominal ultrasound is less sensitive for NAFLD when steatosis accounts for less than 30% of the liver, making it an inferior modality for diagnosing the disease at a 5% liver fat reference value [54]. Therefore, the true prevalence of NAFLD may be underestimated in this study. Third, although several potential confounders have been adjusted for, there are a few critical elements that could not be analyzed, such as genetic factors, diet, and lifestyle, due to the limitations of the original data. Finally, since the population of this study was all Chinese, the externality validation of this study requires results from additional cohort studies.

## 5. Conclusion

The present study demonstrated that elevated METS-IR was independently associated with increased risk of NAFLD development in the nonobese Chinese population and that a dose-response relationship existed. Furthermore, METS-IR may represent a reliable predictor for screening individuals at risk for early NAFLD.

## Figures and Tables

**Table 1:** Characteristics of research subjects.

Variable	METS-IR categories				
	Q1 (18.88-27.66)	Q2 (27.66-30.93)	Q3 (30.94-34.33)	Q4 (34.33-67.05)	*P* value
*N*	2683	2682	2682	2683	
Age (years)	42.62 ± 15.14	43.40 ± 15.01	44.01 ± 15.03	44.56 ± 15.34	<0.001
Sex					<0.001
Women	1331 (49.61%)	1225 (45.67%)	1172 (43.70%)	1111 (41.41%)	
Men	1352 (50.39%)	1457 (54.33%)	1510 (56.30%)	1572 (58.59%)	
BMI (kg/m^2^)	19.17 ± 1.31	21.24 ± 1.18	22.58 ± 1.21	23.53 ± 1.08	<0.001
SBP (mmHg)	114.72 ± 15.61	121.26 ± 16.30	125.84 ± 15.92	129.73 ± 16.62	<0.001
DBP (mmHg)	69.56 ± 9.33	72.83 ± 9.91	75.24 ± 10.10	77.77 ± 10.24	<0.001
TC (mmol/L)	4.57 ± 0.73	4.61 ± 0.74	4.66 ± 0.70	4.64 ± 0.81	<0.001
TG (mmol/L)	0.83 (0.68-1.05)	1.03 (0.82-1.30)	1.26 (0.99-1.61)	1.87 (1.38-2.55)	<0.001
HDL-C (mmol/L)	1.76 ± 0.33	1.56 ± 0.30	1.38 ± 0.25	1.12 ± 0.20	<0.001
FPG (mmol/L)	4.97 ± 0.48	5.14 ± 0.64	5.25 ± 0.82	5.51 ± 1.15	<0.001
LDL-C (mmol/L)	2.13 ± 0.46	2.27 ± 0.47	2.37 ± 0.45	2.34 ± 0.46	<0.001
ALP (U/L)	66.54 ± 24.70	71.24 ± 22.04	74.97 ± 21.85	77.26 ± 21.37	<0.001
GLB (g/L)	29.12 ± 3.77	29.24 ± 3.94	29.32 ± 3.94	29.58 ± 4.23	<0.001
GGT (U/L)	18.00 (14.00-23.00)	20.00 (16.00-27.00)	23.00 (18.00-33.00)	29.00 (21.00-44.00)	<0.001
ALB (g/L)	44.54 ± 2.80	44.53 ± 2.84	44.50 ± 2.71	44.57 ± 2.73	0.856
ALT (U/L)	13.00 (11.00-18.00)	15.00 (12.00-20.00)	18.00 (13.00-24.00)	21.00 (15.00-28.00)	<0.001
AST (U/L)	20.00 (17.00-23.00)	21.00 (18.00-25.00)	22.00 (19.00-26.00)	23.00 (20.00-27.00)	<0.001
TP (g/L)	73.66 ± 4.23	73.77 ± 4.17	73.82 ± 4.19	74.15 ± 4.41	<0.001
BUN (mmol/L)	4.39 ± 1.41	4.64 ± 1.45	4.77 ± 1.35	4.80 ± 1.49	<0.001
Cr (*μ*mol/L)	77.13 ± 21.49	83.21 ± 26.57	86.89 ± 21.95	91.02 ± 35.05	<0.001
UA (mmol/L)	247.34 ± 79.22	282.93 ± 84.85	313.48 ± 82.12	341.57 ± 82.96	<0.001

Variables are presented as the mean ± SD or *n* (%) or median (Q1, Q3). Abbreviations: METS-IR: metabolic score for insulin resistance; SBP: systolic blood pressure; BMI: body mass index; DBP: diastolic blood pressure; TC: total cholesterol; FPG: fasting plasma glucose; HDL-C: high-density lipoprotein cholesterol; TG: triglyceride; LDL-C: low-density lipoprotein cholesterol; AST: aspartate aminotransferase; TP: total protein; ALP: alkaline phosphatase; GLB: globulin; ALB: albumin; Cr: creatinine; BUN: blood urea nitrogen; UA: uric acid; NAFLD: nonalcoholic fatty liver disease.

**Table 2:** Regression models of effects of METS-IR on new-onset NAFLD.

Exposure	Crude model		Model 1		Model 2	
	HR (95% CI)	*P* value	HR (95% CI)	*P* value	HR (95% CI)	*P* value
Q1 (18.88-27.66)	Ref.		Ref.		Ref.	
Q2 (27.66-30.93)	4.74 (3.38, 6.65)	<0.001	2.76 (1.95, 3.91)	<0.001	2.69 (1.87, 3.86)	<0.001
Q3 (30.94-34.33)	11.11 (8.06, 15.33)	<0.001	4.56 (3.21, 6.46)	<0.001	4.25 (2.86, 6.30)	<0.001
Q4 (34.33-67.05)	24.34 (17.75, 33.38)	<0.001	7.82 (5.46, 11.21)	<0.001	6.40 (4.06, 10.08)	<0.001
*P* for trend	<0.001		<0.001		<0.001	

Crude model adjusted for none. Model 1 adjusted for age, sex, and BMI. Model 2 adjusted for the variables in Model 1 plus ALP, TG, GGT, HDL-C, ALT, BUN, AST, Cr, UA, FPG, SBP, LDL-C, and DBP. Abbreviations: CI: confidence interval; HR: hazard ratio. Other abbreviations as in Table 1.

**Table 3:** The results of two-piecewise linear regression model.

Outcome	HR (95% CI)	*P* value
Inflection point of METS-IR	36	
METS-IR < 36	1.31 (1.23, 1.41)	<0.001
METS-IR ≥ 36	1.04 (0.98, 1.10)	0.169
Log-likelihood ratio test		<0.001

Adjusted: age, sex, BMI, ALP, TG, GGT, HDL-C, ALT, BUN, AST, Cr, UA, FPG, SBP, LDL-C, and DBP. Abbreviations as in Tables 1 and 2.

**Table 4:** The value that METS-IR improved risk stratification of NAFLD according to C-index, NRI, and IDI.

	C-index		NRI (category free)		IDI	
	Est. (95% CI)	*P* value	Est. (95% CI)	*P* value	Est. (95% CI)	*P* value
Clinical risk factors	0.719 (0.707, 0.731)		Ref.		Ref.	
Clinical risk factors+METS-IR	0.771 (0.761, 0.781)	<0.001	0.276 (0.247, 0.303)	<0.001	0.068 (0.057, 0.080)	<0.001

Clinical risk factors: BMI, FPG, TC, TG, HDL-C, LDL-C, and GGT. Abbreviations: IDI: integrated discrimination improvement; NRI: net reclassification index. Other abbreviations as in Tables 1 and 2.

## Data Availability

The dataset can be obtained by downloading from the Dryad database (doi:10.5061/dryad.1n6c4).

## Supplementary Materials

Supplementary Materials Supplementary Table 1: collinearity diagnostic steps. Supplementary Table 2: association between METS-IR and new-onset NAFLD when analyzing the METS-IR as a continuous variable.
