# Supplementary material for: Dose-Response Associations of Metabolic Score for Insulin Resistance Index with Nonalcoholic Fatty Liver Disease among a Nonobese Chinese Population: Retrospective Evidence from a Population-Based Cohort Study
Source: Dis Markers. 2022 Feb 23;2022:4930355. doi: 10.1155/2022/4930355 (PMC8890856; doi:10.1155/2022/4930355)
Supplement: Supplementary Materials — Supplementary Table 1: collinearity diagnostic steps. Supplementary Table 2: association between METS-IR and new-onset NAFLD when analyzing the METS-IR as a continuous variable. [file 4930355.f1.docx]

Supplementary Table 1: Collinearity diagnostics steps.

|  | Step 1 | Step 2 | Step 3 |
| --- | --- | --- | --- |
| METS-IR | 46.5 | 46.5 | 3.1 |
| Sex | 1 | 1 | 1 |
| Age | 1.1 | 1.1 | 1.1 |
| ALP | 1.2 | 1.2 | 1.2 |
| GGT | 1.4 | 1.4 | 1.4 |
| ALT | 3.2 | 3.2 | 3.2 |
| AST | 3.2 | 3.2 | 3.2 |
| TP | 19451219.4 | NA | NA |
| ALB | 8262027.1 | NA | NA |
| GLB | 16988033.6 | NA | NA |
| BUN | 1.4 | 1.4 | 1.4 |
| Cr | 1.4 | 1.4 | 1.4 |
| UA | 1.5 | 1.5 | 1.5 |
| FPG | 1.6 | 1.6 | 1.2 |
| TC | 6.3 | 6.3 | NA |
| TG | 5.4 | 5.4 | 1.7 |
| HDL-C | 10.7 | 10.7 | 2.1 |
| LDL-C | 4.6 | 4.6 | 1.1 |
| BMI | 19.3 | 19.3 | 1.3 |
| SBP | 2.4 | 2.4 | 2.4 |
| DBP | 2.1 | 2.1 | 2.1 |

VIF: variance inflation factors. Other abbreviations as in Table 1.

VIF = 1/(1-R^2^). VIF step-by-step screening method: Calculate the VIF of each variable. If the maximum VIF value ≥5, remove the variable with the maximum VIF value.

Supplementary Table 2: Association between METS-IR and new-onset NAFLD when analysing the METS-IR as continuous variable.

| METS-IR (per 1 SD increase) | Coefficient | SE | Z | HR | 95% low | 95% up | P value |
| --- | --- | --- | --- | --- | --- | --- | --- |
| Crude model | 0.71 | 0.02 | 41.49 | 2.02 | 1.96 | 2.10 | <0.001 |
| Model 1 | 0.47 | 0.03 | 16.56 | 1.59 | 1.51 | 1.69 | <0.001 |
| Model 2 | 0.29 | 0.13 | 2.29 | 1.34 | 1.04 | 1.73 | 0.022 |

Crude model adjusted for none.

Model 1 adjusted for age, sex, and BMI.

Model 2 adjusted for the variables in Model 1 plus ALP, TG, GGT, HDL-C, ALT, BUN, AST, Cr, UA, FPG, SBP, LDL-C, and DBP.

Abbreviations as in Table 1 and 2.
